# Global prevalence of malnutrition in older adults: A comprehensive systematic review and meta-analysis

**DOI:** 10.1016/j.puhip.2025.100583

**Published:** 2025-01-10

**Authors:** Nader Salari, Niloofar Darvishi, Yalda Bartina, Fatemeh Keshavarzi, Melika Hosseinian-Far, Masoud Mohammadi

**Affiliations:** a Department of Biostatistics, School of Health, Kermanshah University of Medical Sciences, Kermanshah, Iran; b Department of Psychiatric Nursing, Faculty of Nursing School, Tehran Medical Sciences, Islamic Azad University Science and Research Branch, Tehran, Iran; c Department of Translation Studies, Faculty of Literature, Istanbul University, Istanbul, Turkey; d Student Research Committee, Kermanshah University of Medical Sciences, Kermanshah, Iran; e Department of Food Science & Technology, Ferdowsi University of Mashhad (FUM), Iran; f Research Center for Social Determinants of Health, Jahrom University of Medical Sciences, Jahrom, Iran

**Keywords:** Malnutrition, Nutrition, Elderly, Prevalence, Meta-analysis, World

## Abstract

**Objectives:**

Early detection and management of malnutrition is essential for the general health and well-being of the elderly. Various studies have reported different types of malnutrition prevalence in the elderly. the present study was aimed to determine the prevalence of malnutrition in the world’ elderly through conducting a systematic review study and meta-analysis.

Study Design: systematic review and meta-analysis.

**Methods:**

In this review study, data was extracted by searching in national and international databases of SID, MagIran, Google scholar, ScienceDirect, Scopus, PubMed and Web of Science (WoS) without time limit until August 25, 2023. For analysis, Begg and Mazumdar test at a significance level of 0.1 and the corresponding Funnel plot were used. Data analysis was performed with Comprehensive Meta-Analysis software (Version 2).

**Results:**

In the review of 98 studies with a total sample size of 79976, the prevalence of malnutrition in the world's elderly was obtained as 18.6 % (95 % confidence interval: 16.4-21.1 %), so that the highest prevalence of malnutrition was in the elderly of Africa with 35.7 %, followed by the America with 20.3 %. According to the subgroup analysis regarding the indicators of malnutrition in the elderly, the highest prevalence of malnutrition in the elderly was obtained as 39.9 % according the NRS-2002 index.

**Conclusion:**

Therefore, in addition to raising awareness among families about malnutrition in the elderly and its negative effects on the quality of life of the elderly, it is necessary to take the necessary measures to provide more care for the elderly and also to pay serious attention to the importance of nutrition during old age.

## Abbreviations

GNRI: Geriatric Risk Index
MNA: Mini Nutritional Assessment
MNA-SF: Mini Nutritional Assessment Screening Form
MUST: Malnutrition Universal Screening Tool
MGA: Mental Global Assessment
NRS 2002: Nutritional Risk Screening 2002
WHO: World Health Organization
WoS: Web of Science
STROBE: Strengthening the Reporting of Observational studies in Epidemiology
PRISMA: Preferred Reporting Items for Systematic Reviews and Meta-Analysis

## 1 Background

The elderly are defined as people with a calendar age of 60 years or older. This age group is of particular importance for a variety of reasons. One of these reasons is that the number of older people in the world has been increasing in recent decades. In 2014, the increase in the number of people in this group was three times more than the increase in the total population, and it is expected that the population of this age group will reach more than two billion by 2050 [1,2].

Aging is associated with a wide range of long-term illnesses such as chronic illness, cognitive problems, physical weakness, anorexia, and chewing and swallowing problems which can disrupt the nutritional balance [3]. Nutritional problems include difficulty in chewing, refusing food, and changes in body composition, such as unwanted weight loss and rapid loss of muscle mass [4]. Physical activity decreases with aging that results in receiving fewer calories and reduced consumption of essential nutrients. In addition, older people may change their eating habits for health, social, or financial reasons [5].

Malnutrition in the elderly is defined as a “defective or inadequate nutritional status” characterized by inadequate diet, poor appetite, loss of muscle strength, and weight loss [2]. Malnutrition is caused by inadequate consumption or nutrition which causes various harmful effects such as loss of muscle strength and impaired body defenses [6].

It has been well established among researchers and health care professionals that the elderly are at increased risk of malnutrition [7]. The prevalence of malnutrition is increasing among elderly [8]. Drug use, loneliness, poor oral health, poor quality of life, chronic diseases and frequent hospitalizations affect the health of the elderly and expose them to a higher risk of malnutrition and the risks resulted from malnutrition [9]. Malnutrition can have serious consequences, intensify disease progression, reduce immune function, increase the risk of infection, delay recovery, and prolong the period of hospitalization [10].

Effective prevention and treatment of malnutrition depends on accurate diagnosis. Nutrition screening identifies people who are at risk for malnutrition. Various malnutrition screening tools are used in practice [11]. Some of these tools are based on biochemical and clinical indicators such as Geriatric Risk Index (GNRI). Others are related to anthropometry, mobility, cognitive status and self-perception of health and nutrition such as Mini Nutritional Assessment (MNA) [12] and its shorter version, the Mini Nutritional Assessment Screening Form (MNA-SF) as well as the Malnutrition Universal Screening Tool (MUST) which are the most widely used and effective tools for assessing nutritional status in the elderly [13], while other tools are based on data related to medical, clinical, and patient history and the Mental Global Assessment (MGA) and Nutritional Risk Screening 2002 (NRS 2002) [12]. Malnutrition is assessed by the BMI using the criteria of the World Health Organization (WHO) [14].

In a survey on people over 60 years old in Khorasan Razavi province, Iran, among 1962 elderly, the prevalence of malnutrition based on MNA criteria is 12 % in all elderly, and in women and men is 13.1 % and 10.79 %, respectively [15]. The same index in the elderly of Taiwan urban society showed that 31.71 % of women and 50 % of men suffered from malnutrition [16]. The results of this study showed that the assessment of malnutrition should be performed in the elderly residents in the community [16]. Another survey conducted on 2076 patients aged 65 years and older who were admitted to two rehabilitation hospitals in southeast Sydney and the Ilawara area of Australia showed that the prevalence of malnutrition among them was 32.8 % according to the MNA criteria [17]. The results of a cross-sectional study to determine the prevalence and factors associated with malnutrition among the elderly living in Sri Lanka showed that the prevalence of malnutrition was 12.5 % according to this criterion and, the related factors in this study can help public health professionals to take the necessary interventions to improve the nutritional status of this population [18]. The results of a study on the elderly in the Liguria region in Italy showed that the percentage of malnutrition in the male and female elderly, and in total, were 4.5 %, 1.4 % and 3.4 %, respectively [19]. The results of this study also showed that improving the nutritional status of people living in the community can be used as an effective method to prevent adverse health events such as hospitalization and readmission [19]. Timely identification and management of malnutrition and food insecurity are essential for public health and the health of the elderly [20]. The results of a survey on 1030 elderly people in Turkey showed that malnutrition was 18 % in older women, 20.25 % in older men and 19 % in the elderly as a whole. In this study, age, depression, BMI, and educational status were independently associated with malnutrition in the elderly [21].

Considering the effect of various factors on the prevalence of malnutrition in the elderly and the lack of general statistics in this regard around the world as well as different climatic, economic, cultural and health conditions in the world, in this study it was decided to reach general statistics on the prevalence of malnutrition in the elderly around the world that can be led to an approach to more detailed planning to reduce the effects of malnutrition in the elderly and improve their quality of life through reviewing the studies conducted in this field and statistical analysis of results.

## 2 Methods

### 2.1 Search method

The present study was conducted to determine the prevalence of malnutrition in the elderly worldwide through systematic review and meta-analysis. To collect data in this study, the international and Persian databases of Scopus, Web of science, PubMed, SID, Magiran Google Scholar Science Direct, without time limit until August 25, 2023, were used. The search process in the above-mentioned databases was performed using the keywords “Prevalence, Malnutrition, Elderly, Older adult” and their possible combination in international and Persian databases.

Keywords were extracted from the Medical Subject Headings (MeSH) database and the research question based on PICO was as follows: the studied population (P): the total older adult population in the world, Intervention (I): without intervention, Comparison (C): older adults with malnutrition versus older adults without malnutrition, and Outcomes (O): prevalence of malnutrition in older adults.

A list of titles of all the remaining articles was prepared by the researchers of this study in order to get qualified articles by evaluating the articles in this list. In the first stage, i.e. screening, the titles and abstracts of the remaining articles were carefully studied and irrelevant articles were removed based on the inclusion and exclusion criteria. In the second stage, i.e., the evaluation of the suitability of the studies, the full text of the possible relevant articles remaining from the screening stage was examined based on the inclusion and exclusion criteria, and irrelevant studies were also eliminated in this stage.

PubMed search strategy: (prevalence[Title/Abstract] OR outbreak[Title/Abstract]) AND (Malnutrition[Title] OR Nutritional Deficiency[Title/Abstract] OR Undernutrition[Title/Abstract] OR Malnourishment[Title/Abstract]) AND (Elderly[Title/Abstract] OR aged[Title/Abstract] OR Older adult[Title]) AND (Malnutrition[Title] AND Elderly[Title]) OR (Nutritional Deficiency[Title] AND Older adult[Title]) OR (prevalence[Title] AND Elderly Malnutrition[Title]) NOT (systematic review[Title])

### 2.2 Inclusion and exclusion criteria

Criteria for including articles in the study include: 1- Studies have reported the prevalence of malnutrition in the elderly based on the WHO definition, which refers to deficiencies, excesses, or imbalances in an individual's intake of energy and/or nutrients [22]. However, in this study, we considered nutrient deficiencies in the elderly. cross-sectional studies, population based study.

Criteria for excluding articles in the study include: case control studies, case report, interventional studies, letter to editor studies, studies for which the full text is not available, studies irrelevant to the study subject, cohort studies.

### 2.3 Assessment of quality

To validate and evaluate the quality of articles (i.e., validity of methodology and results), a checklist appropriate to the type of study was used. The STROBE checklist is commonly used to criticize and qualitatively evaluate observational studies such as the present study. the maximum score obtained from the quality assessment in the STROBE checklist will be 32, and considering the score of 16 as the cut-off point, articles with scores of 16 and above were considered as articles with good and average methodological quality, and articles with score below 16 were considered as poor in methodological quality and were thrust excluded from the study.

### 2.4 Statistical analysis

In order to examine heterogeneity in the reviewed studies, the I^2^ test was used and to investigate the publication bias and regarding the high volume of samples included in the study, the Begg and Mazumdar test and its corresponding Funnel plot were used at a significance level of 0.1. Data analysis was performed using Comprehensive Meta-Analysis software (Version 2). Also, to investigate the factors affecting heterogeneity in studies, meta-regression tests were used to examine the sample size, year of study and age of the samples examined in the studies, as well as subgroup analysis in the study by continents and malnutrition indicators in the elderly.

## 3 Results

**Fig. 1:**
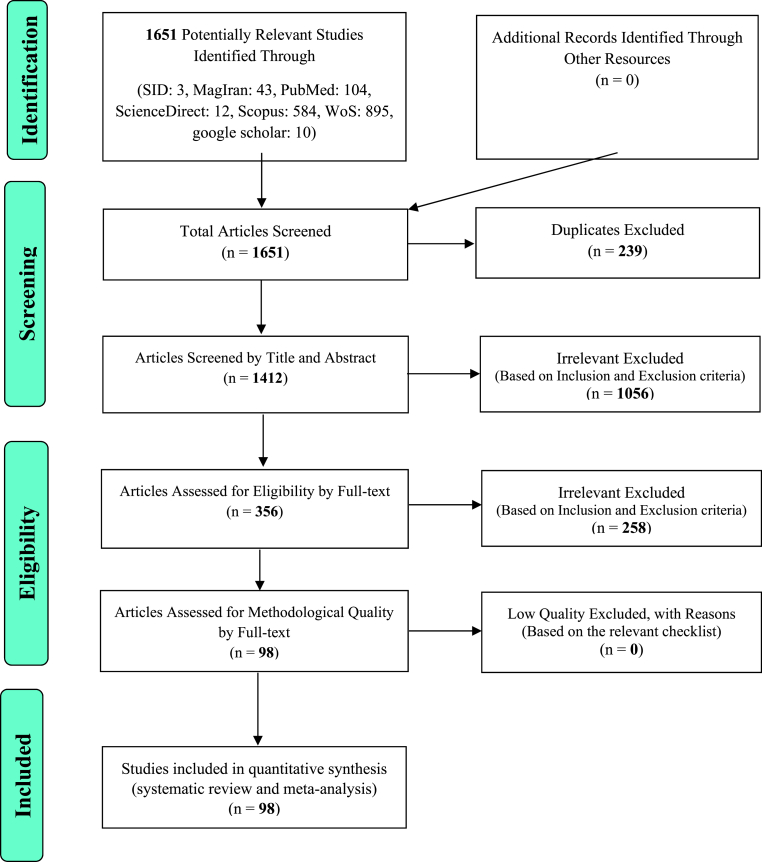
The flowchart on the stages of including the studies in the systematic review and meta-analysis (PRISMA 2009(.

In this study, systematic review and meta-analysis of data from studies on the prevalence of malnutrition of elderly in the world were systematically reviewed according to PRISMA guidelines. Based on the initial search in the intended database, 1651 possible related articles were identified and transferred to the information management software (EndNote). From a total of 1651 identified studies, 239 were repetitive and therefore were excluded. In the screening phase, form 1412 studies, 1056 articles were removed by studying the title and abstract based on inclusion and exclusion criteria. In the competency assessment stage, form the remaining 356 studies, 258 articles were removed by studying the full text of the articles based on inclusion and exclusion criteria due to irrelevance. In the qualitative evaluation stage, by studying the full text of the article and based on the score obtained from the STROBE checklist, no study was excluded from the remaining 98 studies; therefore, 98 articles were finally entered into the final analysis (Fig. 1).

The results of a systematic review of studies were reported in Table 1 according to the review indicators of malnutrition prevalence and the country in which the study was conducted. The lowest and highest sample size were respectively related to the studies of Abdulan et al. (2019) (n = 81) [23] and Wolters et al. (2019) (5956 people) [8]. The characteristics of the studies qualified to be included in the meta-analysis are given in Table 1 (Table 1).

**Table 1:** Information related to the studies entered into the meta-analysis.

Sample number	First author	Year of publication	Place of study (country)	Place of study (continent)	Evaluation criteria	cutoff point	Total sample size	Total prevalence
1	Alhamadan [24]	2019	Saudi Arabia	Asia	MNA	below 17points	2045	24
2	Althobaiti [2]	2019	Saudi Arabia	Asia	MNA	below 17points	152	58
3	Alzahrani [25]	2017	Saudi Arabia	Asia	MNA-SF	below 12points	248	118
4	Abdulan [23]	2019	Romania	Europe	MNA	below 17points	81	17
					SGA	STAGE(B) AND STAGE(C)	81	11
5	Araújo dos Santos [26]	2015	Brazil	America	SGA	STAGE(B) AND STAGE(C)	96	42
6	Adams [27]	2008	Australia	Australia	MNA	below 17points	100	30
7	Boulos [28]	2016	Lebanon	Asia	MNA	below 17points	1200	96
8	Bakker [29]	2018	Netherlands	Europe	20 > BMI	Below 20 points	1022	49
9	Chang, C. C [14]	2011	Taiwan	Asia	18.5> BMI	below 18.5 points	101	16
10	Chang, S. F [16]	2017	Taiwan	Asia	MNA-SF	below 12points	432	132
11	Chen [30]	2012	Malaysia	Asia	18.5> BMI	below 18.5 points	236	41
12	Cuerda [31]	2016	Spain	Europe	MNA	below 17points	1103	107
13	Charlton [17]	2010	Australia	Australia	MNA	below 17points	2076	680
14	Damayanthi [18]	2018	Sri Lanka	Asia	MNA-SF	below 12points	81	18
15	de Bustamante [32]	2018	Spain	Europe	18.5> BMI	below 18.5 points	509	87
16	Donini [13]	2016	Italy	Europe	MNA	below 17points	246	51
17	Donini [33]	2013	Italy	Europe	MNA	below 17points	718	145
18	Damião [34]	2017	Brazil	America	MNA	below 17points	3047	862
19	Demeny [35]	2015	Australia	Australia	MNA	below 17points	101	25
20	Eglseer [36]	2020	Austria	Europe	MUST	BMI<18,5 and weight loss was considered >10 % body weight over the previous 6 months	3702	793
21	Elia [37]	2005	England	Europe	MUST	BMI<18,5 and weight loss was considered >10 % body weight over the previous 6 months	1155	161
22	Ferrari Bravo [19]	2018	Italy	Europe	MNA-SF	below 12points	821	28
23	Ghimire [38]	2018	Nepal	Asia	MNA	below 17points	289	29
24	Geurden [39]	2015	Belgium	Europe	NRS-2002	NRS ≥3	208	107
25	Grammatikopoulou [20]	2019	Greece	Europe	MNA	below 17points	211	111
26	Gruber [40]	2020	Germany	Europe	MNA	below 17points	92	6
27	Gunduz [21]	2015	Turkey	Europe	MNA	below 17points	1030	196
28	Gaskill [41]	2008	Australia	Australia	SGA	STAGE(B) AND STAGE(C)	346	169
29	Hanger [42]	1999	Netherlands	Europe	CMAM	below 5 th	85	7
30	Harris [43]	2008	England	Europe	MNA	below 17points	100	2
31	Isenring [44]	2013	Australia	Australia	MNA-SF	below 12points	254	10
32	Joosten [45]	1999	Belgium	Europe	MNA	below 17points	151	10
33	Keshavarzi [46]	2014	Iran	Asia	MNA	below 17points	447	158
34	Krishnamoorthy [47]	2018	India	Asia	MNA	below 17points	279	50
35	Kucuk [48]	2017	Turkey	Asia	MNA	below 17points	308	88
36	Komici [49]	2019	Italy	Europe	MNA	below 17points	174	21
37	Krzyminska [50]	2016	Poland	Europe	MNA	below 17points	4979	1837
38	Kvamme [51]	2011	Norway	Europe	MUST	BMI<18,5 and weight loss was considered >10 % body weight over the previous 6 months	3111	222
39	Kvamme [52]	2015	Norway	Europe	MUST	BMI<18,5 and weight loss was considered >10 % body weight over the previous 6 months	1521	122
40	Keller [53]	1993	Canada	America	20 > BMI	Below 20 points	200	103
41	Li, T [54]	2020	China	Asia	MNA	below 17points	182	96
42	Lacau St Guily [55]	2018	France	Europe	18.5> BMI	below 18.5 points	578	552
43	Liguori [56]	2018	Italia	Europe	MNA	below 17points	473	70
44	Lindroos [57]	2014	Finland	Europe	MNA	below 17points	1466	195
45	Lara-Pulido [58]	2012	Mexico	America	MNA	below 17points	769	54
46	Lecheta [4]	2017	Brazil	America	MNA	below 17points	96	5
47	Mathew [59]	2016	India	Asia	MNA	below 17points	190	37
48	Miao [60]	2019	China	Asia	MNA	below 17points	425	249
49	Mokhber [61]	2011	Iran	Asia	MNA	below 17points	1565	172
50	Madeira [62]	2019	Spain	Europe	MNA	below 17points	1186	570
51	Mitrache [63]	2001	Switzerland	Europe	Biochemical evidence	Biochemical evidence of malnutrition	186	46
52	Morrone [64]	2011	Italia	Europe	MNA	below 17points	718	145
53	Manson [65]	1991	USA	America	MNA	below 17points	100	48
54	Nazemi [66]	2015	Iran	Asia	MNA	below 17points	263	27
55	Ning [67]	2020	China	Asia	MNA	below 17points	2323	416
56	Nogay [68]	2012	Turkey	Asia	MNA-SF	below 12points	473	37
57	Norazman [69]	2020	Malaysia	Asia	MNA-SF	below 12points	301	99
58	Nelson [70]	1993	USA	America	20 > BMI	Below 20 points	100	39
59	Naidoo [71]	2015	Africa	Africa	MNA-SF	below 12points	984	351
60	Orlandoni [72]	2017	Italia	Europe	MUST	BMI<18,5 and weight loss was considered >10 % body weight over the previous 6 months	284	70
61	Paris [73]	2013	Spain	Europe	MNA	below 17points	1098	430
62	Poulia [12]	2012	Greece	Europe	MUST	BMI<18,5 and weight loss was considered >10 % body weight over the previous 6 months	248	129
					SGA	STAGE(B) AND STAGE(C)	248	43
					MNA-SF	below 12points	248	88
					NRS-2002	NRS ≥3	248	73
					NRI	below 98points	248	37
					GNRI	GNRI< 92	248	22
63	Rashid [74]	2020	India	Asia	MNA-SF	below 12points	235	109
64	Raposeiras-Roubín [75]	2020	Spain	Europe	CONUT	CONUT>1	4724	2036
65	Ribeiro [76]	2011	Brazil	America	MNA	below 17points	236	13
66	Rodríguez-Tadeo [77]	2012	Mexico	America	MNA	below 17points	760	61
67	Simsek [78]	2013	Turkey	Asia	MNA	below 17points	650	18
68	Su [79]	2020	Japan	Asia	MNA-SF	below 12points	294	69
69	Sanz París [80]	2013	Spain	Europe	MNA	below 17points	1098	232
70	Seljak [81]	2020	Slovenian	Europe	MNA-SF	below 12points	207	42
71	Serrano-Urrea [82]	2013	Spain	Europe	MNA	below 17points	895	25
72	Slavikova [83]	2018	Czech	Europe	MNA	below 17points	254	27
73	Sahin [84]	2016	USA	America	MNA	below 17points	257	22
74	Sharma [85]	2017	Australia	Australia	SGA	STAGE(B) AND STAGE(C)	650	18
75	Tsai [86]	2008	Taiwan	Asia	MNA	below 17points	2890	58
76	Tagliaferri [87]	2019	Italy	Europe	MNA-SF	below 12points	773	124
77	Ulger [88]	2013	Turkey	Asia	MNA-SF	below 12points	534	85
78	Vafaei [89]	2013	Iran	Asia	MNA	below 17points	370	14
79	Vedantam [90]	2010	India	Asia	MNA	below 17points	227	32
80	van der Sijp [91]	2018	Netherlands	Europe	MNA-SF	below 12points	437	55
81	Vanderwee [92]	2010	Belgium	Europe	MNA	below 17points	2329	754
82	Verbrugghe [93]	2013	Belgium	Europe	MNA	below 17points	886	191
83	Volkert [94]	2011	Germany	Europe	MNA	below 17points	382	94
84	Wong [95]	2019	China	Asia	MNA	below 17points	613	179
85	Woo [96]	2005	China	Asia	18.5> BMI	below 18.5 points	1820	379
86	Westergren [97]	2015	Sweden	Europe	SCREEN II	below 53points	465	30
87	Win [98]	2017	USA	America	MNA-SF	below 12points	2252	344
88	Wolters [8]	2019	New Zealand	Australia	20 > BMI	Below 20 points	5956	155
89	Yoshimura [99]	2013	Japan	Asia	MNA-SF	below 12points	274	77
90	Zainudin [100]	2019	Malaysia	Asia	MNA-SF	below 12points	413	106
91	Zenthofer [101]	2015	Germany	Europe	20 > BMI	Below 20 points	255	222
92	Zhang [22]	2019	USA	America	MNA-SF	below 12points	454	190

**Fig. 2:**
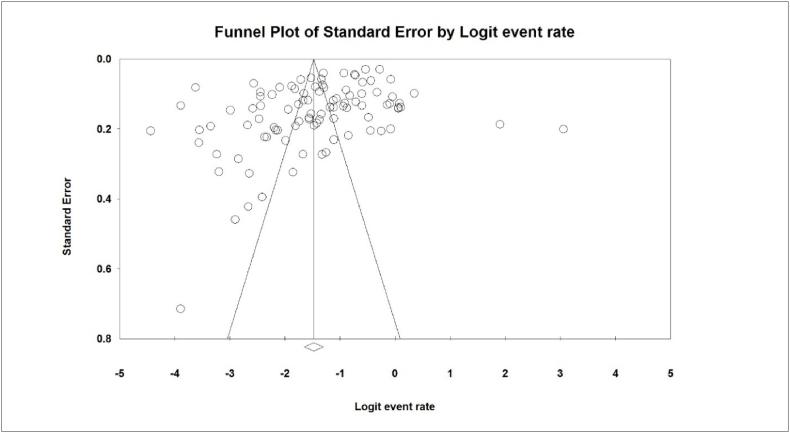
Funnel plot indicating the results related to the prevalence of malnutrition in the world's elderly.

According to the test results (I^2^: 98.8) and considering the heterogeneity of the selected studies, a stochastic effects model was used to combine studies and estimate the prevalence. The reason for heterogeneity between studies can be due to differences in sample size, year of study or place of study. The publication bias of the results of malnutrition prevalence of the elderly in the world by funnel diagram and Begg and Mazumdar test at a significance level of 0.1 showed no bias in the present study (P = 0.112) (Fig. 2).

**Fig. 3:**
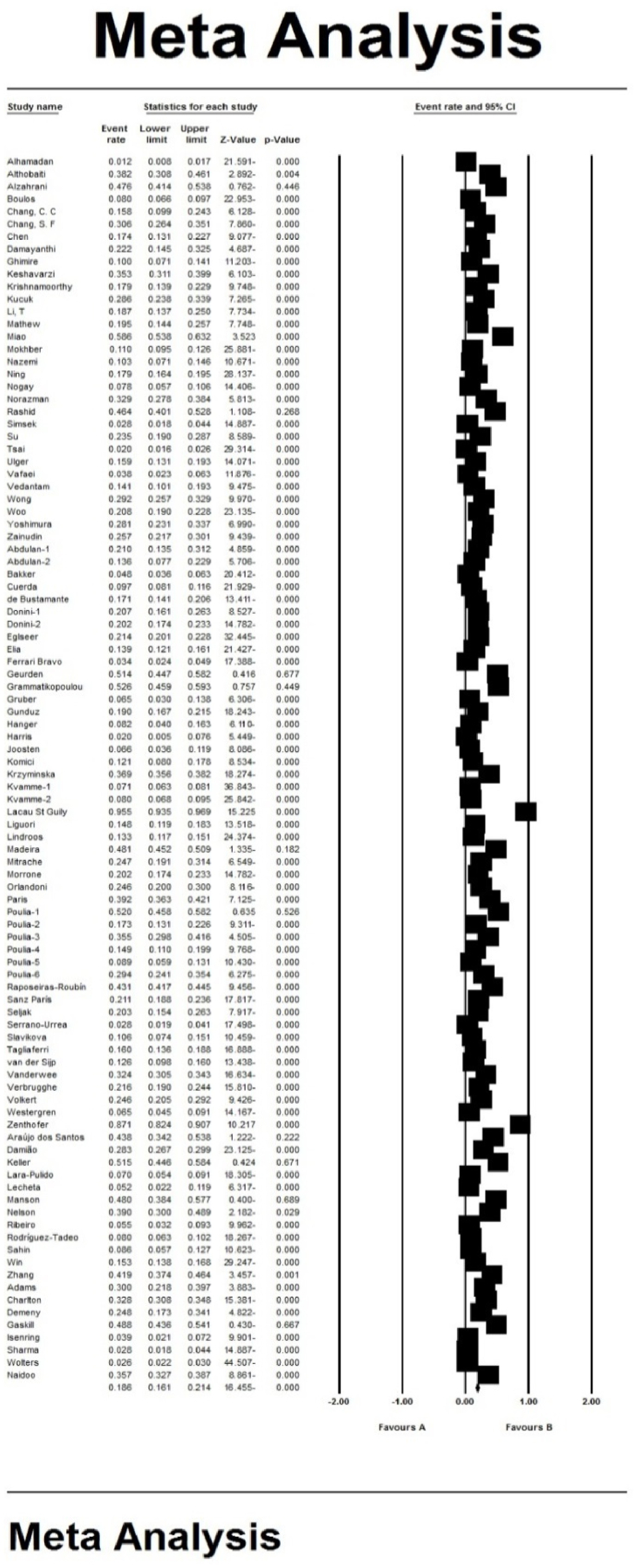
Prevalence of malnutrition in the world's elderly and 95 % confidence interval based on the random effects model.

In the review of 98 studies (31 studies in Asia, 47 in Europe, 1 in Africa, 12 in the America and 7 in Australia) (Abdulan [23] has two data and Poulia [12] has six separate prevalence data.) with a total sample size of 79976, the prevalence of malnutrition in the elderly worldwide was obtained as 18.6 % (95 % confidence interval: 16.4-21.1. The shape of Forrest Plot 3 indicates the overall prevalence in the studied studies, and the midpoint of each line segment of shows the prevalence in each study and diamond shape indicates the prevalence in population for all studies (Fig. 3).

### 3.1 Meta-regression test

**Fig. 4:**
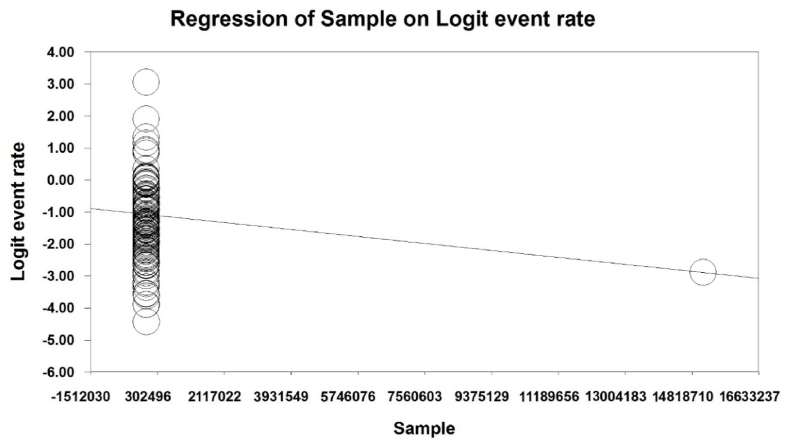
Meta-regression chart of the prevalence of malnutrition in the world's elderly in terms of sample size.

**Fig. 5:**
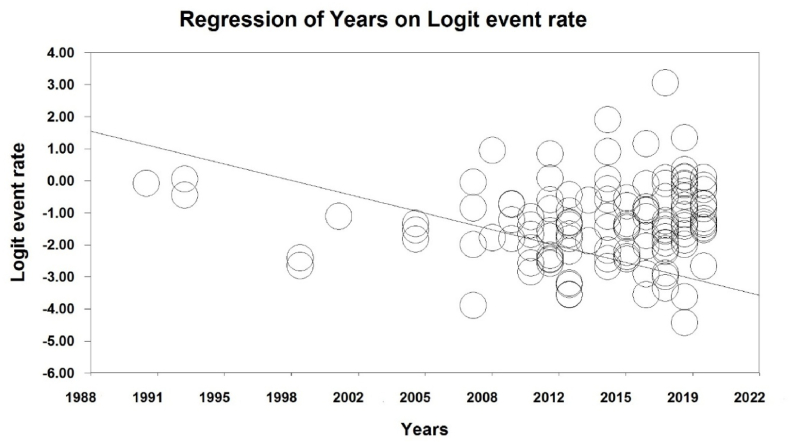
Meta-regression chart of the prevalence of malnutrition in the world's elderly in terms of the year of study.

**Fig. 6:**
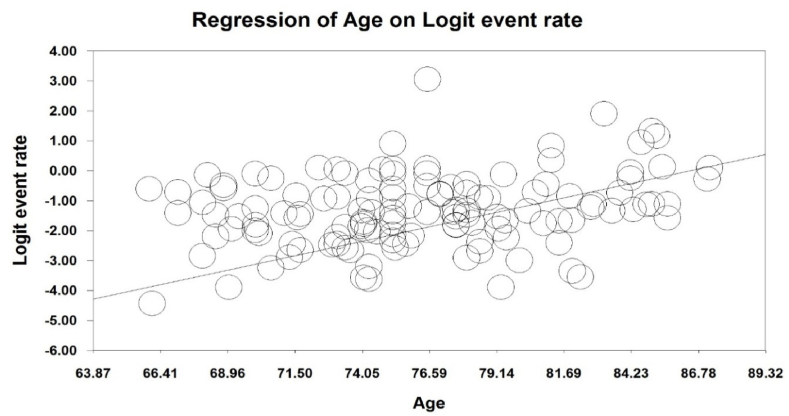
Meta-regression chart of the prevalence of malnutrition in the world's elderly in terms of the age of study participants.

To investigate the effects of potential factors in the heterogeneity of the prevalence of malnutrition in the elderly worldwide, meta-regression was used on three factors: sample size, year of the study and age of the study participants (Fig. 4, Fig. 5, Fig. 6). According to Fig. 4, the prevalence of malnutrition in the world's elderly decreases with an increase in sample size that is statistically significant (P < 0.05). In Fig. 5, it was also reported that the prevalence of malnutrition in the world's elderly decreases with an increase in the year of study that this difference was also statistically significant (P < 0.05), while the results reported in Fig. 6 show that with increasing the age of participants in the study, the prevalence of malnutrition in the elderly increases, which this difference was also statistically significant (P < 0.05).

### 3.2 Analysis of subgroups

In Table 2 which reports the prevalence of malnutrition in the world's elderly in terms of different continents, these changes are reported in Asia, Europe, Africa, America and Australia. Based on the results in this table, the highest prevalence of malnutrition in the elderly of Africa was 35.7 % (95 % confidence interval: 32.7-38.7 %) and 20.3 % in America (95 % confidence interval: 13.7–29 %) (Table 2).

**Table 2:** Prevalence of malnutrition in the world's elderly in terms of different continents.

Continents	Number of articles	Sample Size	I^2^	Begg and Mazumdar Test	Prevalence % (95 % CI)
Asia	31	19860	98.6	0.109	18.3 (95 % CI: 14.8-22.3)
Europe	47	41282	98.9	0.804	19.8 (95 % CI: 16.2–24)
America	12	8367	98.1	0.631	20.3 (95 % CI: 13.7–29)
Africa	1	984	0	–	35.7 (95 % CI: 32.7-38.7)
Australia	7	9483	99.5	0.543	13.4 (95 % CI: 4.3-34.8)

According to the results of studies conducted on the prevalence of malnutrition in the world's elderly, a subgroup analysis was also performed in accordance with the indicators through which the malnutrition in the elderly has been examined, which shows that the highest prevalence of malnutrition in the elderly was obtained as 39.9 % (95 % confidence interval: 21.1-56.7 %) based on the NRS- 2002 and BMI index was obtained as <18.5 with 35 % (95 % confidence interval: 13.1-65.7 %) (Table 3).

**Table 3:** Prevalence of malnutrition in the world's elderly of the world according to review indicators of malnutrition.

Index type	Number of articles	Sample Size	I^2^	Begg and Mazumdar Test	Prevalence % (95 % CI)
**BMI<18.5**	5	3244	99.1	0.102	35 (95 % CI: 13.1-65.7)
BMI<20	5	7533	99.6	0.707	26.5 (95 % CI: 17.1-50.1)
MNA	50	3978	98.7	0.121	16.9 (95 % CI: 13.6-20.7)
MNA-SF	19	11565	97.4	0.290	22.3 (95 % CI: 17.6-27.9)
MUST	6	10021	99.1	0.763	17.8 (95 % CI: 10.2-29.2)
NRS-2002	2	456	95.5	–	39.9 (95 % CI: 21.1-56.7)
SGA	5	976	95.5	0.220	33.2 (95 % CI: 19.9-49.8)
GNRI	1	465	100	0	6.5 (95 % CI: 4.5-9.1)

## 4 Discussion

Our findings indicate high prevalence of malnutrition in the elderly [50]. The prevalence of malnutrition in Chinese hospitalized elderly is between 40.9 % and 58.6 % [60]. Similarly, a cross-sectional study in India and a survey in older patients in Spain, a study on the prevalence and factors associated with malnutrition in elderly patients with malnutrition in the United States, a cross-sectional study in South Africa, and a study related to nutrition issues in the elderly living in Australia, all described the high prevalence of malnutrition in the elderly [22,41,71,74,102].

In our study, the prevalence of malnutrition was between 21.1 % and 56.7 % according to NRS-2002 index. In a study by Geurden et al., the prevalence of malnutrition was 51.4 % according to the NRS-2002 index [39]. In our study, the prevalence of malnutrition based on BMI <18.5 and BMI <20 and MNA and MNA-SF was 35 %, 26.5 %, 16.9 % and 22.3 %, respectively. Similarly, a study in Malaysia reported the prevalence of malnutrition in the elderly equal to 19.9 % based on BMI <18.5 [30]. Moreover, a study among the elderly admitted to Dutch hospitals reported the prevalence of malnutrition as 69.8 based on BMI index <20 [103]. In another study conducted among the elderly in Italy, the prevalence of malnutrition based on the MNA index was reported as 20.73 % [13]; and in a study among the elderly in Japan, the prevalence of malnutrition based on the MNA-SF index was reported as 23.46 % [79].

In the present study, the prevalence of malnutrition based on MUST, SGA and GNRI indices is 17.8 %, 33.2 % and 6.5 %, respectively. In another study by Orlandoni et al. in Italy, the prevalence of malnutrition was reported as 24.64 % based on the MUST index [72]. In the study by Araújo dos Santos et al. in Brazil, the prevalence of malnutrition was reported as 43.75 % based on SGA index, [26]. Also, in another study by Nakamura et al. in Japan, the prevalence of malnutrition was reported as 52.58 % based on the GNRI index [104].

There are many factors that can explain the high prevalence of malnutrition. Low body weight, low BMI, physiological anorexia of elderly, decreased physical activity, and muscle mass are among these factors [16]. Also, those who are physically weak and have a higher average age and lower household income are also at risk of malnutrition [69]. Other factors such as decreased sense of taste and smell which may generally occur with aging, reduce appetite and malnutrition [55]. Along with physiological changes, several psychological determinants and environmental changes such as isolation, loneliness, depression and insufficient income affect the consumption of diet and thus nutritional status [55]. Moreover, having an underlying disease in elderly, drug interventions and side effects of drugs can play a role in the development of malnutrition in elderly [22].

The elderly face issues that put their nutritional status at a greater risk. It is clear that these issues need immediate attention [83]. Early detection of the elderly with cancer and risk factors for anaemia and depression provides nutritional interventions that may improve treatment tolerance, quality of life, and survival outcomes [105]. Paying attention to individual health issues and related factors which may affect their eating habits as well as providing appropriate interventions to achieve a desirable and healthy diet are critical in elderly [100].

When comparing the prevalence of malnutrition in elderly on different continents, we found that elderly in Africa, the United States and Europe have the highest prevalence of malnutrition, respectively. We believe that this could be due to differences in economic status, demographic conditions as well as the psychological conditions of elderly and their lifestyles in different countries. For example, older people in Uganda have been described as lacking income and pensions, living in crowded homes, and being overwhelmed with illness [71]. In a study conducted in Brazil, it has been stated that the use of elderly people living in the community rather than hospitalized, as well as appropriate social and psychological support and practical approaches that improve calorie intake, have led to a lower prevalence of malnutrition in elderly [76]. It has also been suggested that Polish elderly with symptoms of depression who suffer from multiple illnesses and anaemia should be monitored and controlled for signs of malnutrition [50]. In this study, Asian and Australian seniors had a lower prevalence of malnutrition, so that the lowest prevalence of malnutrition was in Australia. A study in Iran has also shown that the nutritional status of the elderly is related to education, so that a higher level of education was associated with higher income and a better lifestyle, which in turn leads to a better nutritional status in these older people [15]. In another study conducted in Australia, the prevalence of malnutrition in elderly was very low when compared to similar studies, which was probably due to accurate criteria for inclusion of people in the study and lifestyle of elderly [44].

Comparing the prevalence of malnutrition based on different indicators, we found that the prevalence of malnutrition in the elderly is higher based on NRS-2002 and BMI <18.5 than other indicators. The NRS 2002 was developed to determine who needs nutritional support and may identify more patients at high risk of malnutrition [106]. Poor nutrition is proposed as a problem in long-term care, so body weight should be recorded as the most important nutritional indicator and it is independently associated with aging [96].

In our study, SGA, BMI <20, MNA-SF, and MUST indices are in the next ranks in terms of the prevalence of malnutrition, respectively. SGA is an indicator of the calculation of malnutrition in older patients who naturally have a high prevalence of malnutrition. SGA has been tested and validated in a variety of clinical settings and is considered as a relatively accurate, easy, and rapid tool for estimating the nutritional risk due to the inclusion of information from clinical examination as well as medical history and anthropometrics [12]. In a study conducted to evaluate different indicators of the prevalence of malnutrition, the sensitivity and specificity of MNA-SF index were higher. This finding is reasonable and expected because MNA-SF has been specifically designed for elderly and it is used for all statuses of older people (sick or healthy) [12]. In another study conducted in China, it was suggested that MUST is a useful and very effective screening tool [106].

The prevalence of malnutrition based on GNRI and MNA indices is the lowest, respectively. The specific index of aging, GNRI, is an important tool for screening malnutrition in elderly hospitalized and with rehabilitation care and long-term care [12] that the prevalence of malnutrition is clearly higher in this group of elderly. The MNA tool is assumed to be a “better” screening tool for use in elderly; however, data from some studies may not support this hypothesis [43].

In our study, we found a statistically significant difference in the prevalence of malnutrition with increasing sample size, so that with increasing sample size, the prevalence of malnutrition in the world's elderly decreases. In a study by Ahmed et al. with a sample size of 15121131 elderly, the prevalence of nutrition was equal to 5.29 % [107]. Another study conducted by Win et al. with a sample size of 2252 elderly, the prevalence of malnutrition was equal to 15.27 %. Also, a study by Kucuk et al. with a sample size of 308 elderly, the prevalence of malnutrition was equal to 28.57 %. A high volume of study seems to increase the accuracy of malnutrition diagnosis and reduce error.

In our study, we found a statistically significant difference in the prevalence of malnutrition with the increase in the years of study, so that with increase in years of study, the prevalence of malnutrition in the world's elderly decreases. Moreover, we found a statistically significant difference in the prevalence of malnutrition with increasing age in the elderly, so that with the increase in the age of the elderly, the prevalence of malnutrition in the world's elderly increases. A study by de Guzman et al. showed that malnutrition and its risks are more common in the elderly over 70 years [108]. In another study by Damayanthi et al., the results of univariate analysis showed that higher age, hypertension, smoking and alcohol consumption were significantly associated with the prevalence of malnutrition [18]. Also, an a study by Elia et al., the prevalence of malnutrition in the age groups of 65–74 years, 75–84 years, 85 years and above was statistically significant, so that the prevalence of malnutrition was higher with increase in age [37].

## 5 Conclusion

In this meta-analysis, the prevalence of malnutrition in the elderly worldwide is 18.6 %, which is significantly high. Further research should be conducted to identify the risk factors for malnutrition in the elderly in the socio-cultural and economic fields to develop effective screening strategies and identify and assist the elderly suffered from malnutrition.

## Ethics approval and consent to participate

Ethics approval was received from the ethics committee of deputy of research and technology, Kermanshah University of Medical Sciences (IR.KUMS.REC.1400.509).

## Consent for publication

Not applicable.

## Availability of data and materials

Datasets are available through the corresponding author upon reasonable request.

## Author contribution

NS and ND and FK contributed to the design, MM statistical analysis, participated in most of the study steps. YB and MHF and MM and ND and FK prepared the manuscript. All authors have read and approved the content of the manuscript

## Funding

By Deputy for Research and Technology, Kermanshah University of Medical Sciences (IR) (4000637). This deputy has no role in the study process.

## Declaration of competing interest

The authors declare that they have no known competing financial interests or personal relationships that could have appeared to influence the work reported in this paper.

## References

[1] Badrasawi M. (2019). Malnutrition and its association with functional, cognitive and psychological status among Palestinian older adults in long-term care houses. Educ. Gerontol..

[2] Althobaiti M.M.M. (2019). The prevalence of geriatric malnutrition and its factors in Saudi Arabia. Indo American Journal of Pharmaceutical Sciences.

[3] Khoddam H. (2019). Prevalence of malnutrition among elderly people in Iran: protocol for a systematic review and meta-analysis. Jmir Research Protocols.

[4] Lecheta D.R. (2017). Nutritional problems in older adults with Alzheimer's disease: risk of malnutrition and sarcopenia. Revista De Nutricao-Brazilian Journal of Nutrition.

[5] Guigoz Y., Lauque S., Vellas B.J. (2002). Identifying the elderly at risk for malnutrition. The mini nutritional assessment. Clin. Geriatr. Med..

[6] Maitre I. (2014). Food pickiness in the elderly: relationship with dependency and malnutrition. Food Qual. Prefer..

[7] Volkert D. (2020). Joint action malnutrition in the elderly (MaNuEL) knowledge hub: summary of project findings. Eur Geriatr Med.

[8] Wolters M. (2019). Prevalence of malnutrition using harmonized definitions in older adults from different settings - a MaNuEL study. Clin. Nutr..

[9] Gorji H.A. (2017). The prevalence of malnutrition in Iranian elderly: a review article. Iran. J. Public Health.

[10] Lin W.Q. (2017). The unhealthy lifestyle factors associated with an increased risk of poor nutrition among the elderly population in China. J. Nutr. Health Aging.

[11] Yaxley A., Crotty M., Miller M. (2015). Identifying malnutrition in an elderly ambulatory rehabilitation population: agreement between mini nutritional assessment and validated screening tools. Healthcare (Basel).

[12] Poulia K.A. (2012). Evaluation of the efficacy of six nutritional screening tools to predict malnutrition in the elderly. Clin. Nutr..

[13] Donini L.M. (2016). Mini-nutritional assessment, malnutrition universal screening tool, and nutrition risk screening tool for the nutritional evaluation of older nursing home residents. J. Am. Med. Dir. Assoc..

[14] Chang C.C., Roberts B.L. (2011). Malnutrition and feeding difficulty in Taiwanese older with dementia. J. Clin. Nurs..

[15] Aliabadi M. (2008). Prevalence of malnutrition in free living elderly people in Iran: a cross-sectional study. Asia Pac. J. Clin. Nutr..

[16] Chang S.F. (2017). Frailty is a major related factor for at risk of malnutrition in community-dwelling older adults. J. Nurs. Scholarsh..

[17] Charlton K.E. (2010). Older rehabilitation patients are at high risk of malnutrition: evidence from a large Australian database. J. Nutr. Health Aging.

[18] Damayanthi H. (2018). Prevalence of malnutrition and associated factors among community-dwelling older persons in Sri Lanka: a cross-sectional study. BMC Geriatr..

[19] Ferrari Bravo M. (2018). Assessment of malnutrition in community-dwelling elderly people: cooperation among general practitioners and public health. Iran. J. Public Health.

[20] Grammatikopoulou M.G. (2019). Food insecurity increases the risk of malnutrition among community-dwelling older adults. Maturitas.

[21] Gunduz E. (2015). Malnutrition in community-dwelling elderly in Turkey: a multicenter, cross-sectional study. Med. Sci. Mon. Int. Med. J. Exp. Clin. Res..

[22] Geneva: World Health Organization (2017).

[23] Abdulan I.M. (2019). The predictive value of malnutrition for functional and cognitive status in elderly hemodialysis patients. Int. Urol. Nephrol..

[24] Alhamadan A.A. (2019). Prevalence of malnutrition and its association with activities of daily living in older adults attending primary health care centers: a multistage cross-sectional study. Prog. Nutr..

[25] Alzahrani S.H., Alamri S.H. (2017). Prevalence of malnutrition and associated factors among hospitalized elderly patients in King Abdulaziz University Hospital, Jeddah, Saudi Arabia. BMC Geriatr..

[26] Araújo dos Santos C. (2015). Patient-generated subjective global assessment and classic anthropometry: comparison between the methods in detection of malnutrition among elderly with cancer. Nutr. Hosp..

[27] Adams N.E. (2008). Recognition by medical and nursing professionals of malnutrition and risk of malnutrition in elderly hospitalised patients. Nutr. Diet..

[28] Boulos C., Salameh P., Barberger-Gateau P. (2016). Malnutrition and frailty in community dwelling older adults living in a rural setting. Clin. Nutr..

[29] Bakker M.H. (2018). Are edentulousness, oral health problems and poor health-related quality of life associated with malnutrition in community-dwelling elderly (aged 75 Years and over)? A cross-sectional study. Nutrients.

[30] Chen S.T., Ngoh H.J., Harith S. (2012). Prevalence of malnutrition among institutionalized elderly people in northern peninsular Malaysia: gender, ethnicity and age-specific. Sains Malays..

[31] Cuerda C. (2016). Prevalence of malnutrition in subjects over 65 years of age in the Community of Madrid. the DREAM+65 Study. Nutr. Hosp..

[32] de Bustamante, M.D., et al., Prevalence of malnutrition in a cohort of 509 patients with acute hip fracture: the importance of a comprehensive assessment*.* Eur. J. Clin. Nutr., 20(72): 77-81. 10.1038/ejcn.2017.72 28513623

[33] Donini L.M. (2013). Malnutrition in elderly: social and economic determinants. J. Nutr. Health Aging.

[34] Damião R. (2017). Factors associated with risk of malnutrition in the elderly in south-eastern Brazil. Rev. Bras. Epidemiol.

[35] Demeny D. (2015). Current practices of dietitians in the assessment and management of malnutrition in elderly patients. Nutr. Diet..

[36] Eglseer D., Hoedl M., Schoberer D. (2020). Malnutrition risk and hospital-acquired falls in older adults: a cross-sectional, multicenter study. Geriatr. Gerontol. Int..

[37] Elia M., Stratton R.J. (2005). Geographical inequalities in nutrient status and risk of malnutrition among English people aged 65 y and older. Nutrition.

[38] Ghimire S. (2018). Depression, malnutrition, and health-related quality of life among Nepali older patients. BMC Geriatr..

[39] Geurden B. (2015). The risk of malnutrition in community-living elderly on admission to hospital for major surgery. Acta Chir. Belg..

[40] Gruber M.T. (2020). Association between malnutrition, clinical parameters and health-related quality of life in elderly hospitalized patients with Parkinson's disease: a cross-sectional study. PLoS One.

[41] Gaskill D. (2008). Malnutrition prevalence and nutrition issues in residential aged care facilities. Australas. J. Ageing.

[42] Hanger H.C. (1999). The prevalence of malnutrition in elderly hip fracture patients. N. Z.Med. J..

[43] Harris D.G. (2008). An observational study of screening for malnutrition in elderly people living in sheltered accommodation. J. Hum. Nutr. Diet..

[44] Isenring E., Baker J., Kerr G. (2013). Malnutrition and falls risk in community-dwelling older adults. J. Nutr. Health Aging.

[45] Joosten E., Vanderelst B., Pelemans W. (1999). The effect of different diagnostic criteria on the prevalence of malnutrition in a hospitalized geriatric population. Aging Clin. Exp. Res..

[46] Keshavarzi S., Ahmadi S.M., Lankarani K.B. (2014). The impact of depression and malnutrition on health-related quality of life among the elderly Iranians. Global J. Health Sci..

[47] Krishnamoorthy Y. (2018). Prevalence of malnutrition and its associated factors among elderly population in rural Puducherry using mini-nutritional assessment questionnaire. J. Fam. Med. Prim. Care.

[48] Kucuk E.O., Kapucu S. (2017). Malnutrition in elderly staying in nursing homes. Konuralp Tip Dergisi.

[49] Komici K. (2019). Impact of malnutrition on long-term mortality in elderly patients with acute myocardial infarction. Nutrients.

[50] Krzyminska-Siemaszko R. (2016). Health status correlates of malnutrition in the polish elderly population - results of the Polsenior Study. Eur. Rev. Med. Pharmacol. Sci..

[51] Kvamme J.M. (2011). Risk of malnutrition is associated with mental health symptoms in community living elderly men and women: the Tromso Study. BMC Psychiatr..

[52] Kvamme J.M. (2015). Risk of malnutrition and zinc deficiency in community-living elderly men and women: the Tromso Study. Publ. Health Nutr..

[53] Keller H.H. (1993). Malnutrition in institutionalized elderly - HOW and WHY. J. Am. Geriatr. Soc..

[54] Li T. (2020). Prevalence of malnutrition and analysis of related factors in elderly patients with COVID-19 in Wuhan, China. Eur. J. Clin. Nutr..

[55] Lacau St Guily J. (2018). NutriCancer: a French observational multicentre cross-sectional study of malnutrition in elderly patients with cancer. J Geriatr Oncol.

[56] Liguori I. (2018). Risk of malnutrition evaluated by mini nutritional assessment and sarcopenia in noninstitutionalized elderly people. Nutr. Clin. Pract..

[57] Lindroos E. (2014). CAREGIVER-REPORTED swallowing difficulties, malnutrition, and mortality among older people in assisted living facilities. J. Nutr. Health Aging.

[58] Lara-Pulido A., Guevara-Cruz M. (2012). Malnutrition and associated factors in elderly hospitalized. Nutr. Hosp..

[59] Mathew A.C. (2016). Prevalence and correlates of malnutrition among elderly in an urban area in Coimbatore. Indian J. Publ. Health.

[60] Miao J.P. (2019). Comparison of two malnutrition risk screening tools with nutritional biochemical parameters, BMI and length of stay in Chinese geriatric inpatients: a multicenter, cross-sectional study. BMJ Open.

[61] Mokhber N. (2011). Association between malnutrition and depression in elderly people in Razavi khorasan: a population based-study in Iran. Iran. J. Public Health.

[62] Madeira T. (2019). Malnutrition among older adults living in Portuguese nursing homes: the PEN-3S study. Publ. Health Nutr..

[63] Mitrache C. (2001). Anemia: an indicator for malnutrition in the elderly. Ann. Hematol..

[64] Morrone A. (2011). [Malnutrition in the elderly: clinical features, psychological and social determinants. Preliminary results]. Ann Ig.

[65] Manson A., Shea S. (1991). Malnutrition in elderly ambulatory medical patients. Am. J. Publ. Health.

[66] Nazemi L. (2015). Malnutrition, prevalence and relation to some risk factors among elderly residents of nursing homes in tehran, Iran. Iran. J. Public Health.

[67] Ning H. (2020). Malnutrition and its associated factors among elderly Chinese with physical functional dependency. Publ. Health Nutr..

[68] Nogay N.H., Akinci A.C. (2012). Malnutrition risk and associated factors among elderly people in Turkey. HealthMED.

[69] Norazman C.W., Adznam S.N., Jamaluddin R. (2020). Malnutrition as key predictor of physical frailty among Malaysian older adults. Nutrients.

[70] Nelson K.J. (1993). Prevalence of malnutrition in the elderly admitted to long-term-care facilities. J. Am. Diet Assoc..

[71] Naidoo I. (2015). High risk of malnutrition associated with depressive symptoms in older South Africans living in KwaZulu-Natal, South Africa: a cross-sectional survey. J. Health Popul. Nutr..

[72] Orlandoni P. (2017). Malnutrition upon hospital admission in geriatric patients: why assess it?. Front. Nutr..

[73] Paris A.S. (2013). Malnutrition prevalence in hospitalized elderly diabetic patients. Nutr. Hosp..

[74] Rashid I., Tiwari P., Lehl S.S. (2020). Malnutrition among elderly a multifactorial condition to flourish: evidence from a cross-sectional study. Clinical Epidemiology and Global Health.

[75] Raposeiras-Roubín S. (2020). Impact of malnutrition in the embolic-haemorrhagic trade-off of elderly patients with atrial fibrillation. Europace.

[76] Ribeiro R.S., Rosa M.I., Bozzetti M.C. (1992). Malnutrition and associated variables in an elderly population of Criciúma, SC. Rev. Assoc. Med. Bras..

[77] Rodríguez-Tadeo A. (2012). Malnutrition risk factors among the elderly from the US-Mexico border: the "one thousand" study. J. Nutr. Health Aging.

[78] Simsek H. (2013). Prevalence of food insecurity and malnutrition, factors related to malnutrition in the elderly: a community-based, cross-sectional study from Turkey. European Geriatric Medicine.

[79] Su Y. (2020). Denture wearing and malnutrition risk among community-dwelling older adults. Nutrients.

[80] Sanz París A. (2013). Malnutrition prevalence in hospitalized elderly diabetic patients. Nutr. Hosp..

[81] Seljak B.K. (2020). A multi-center survey on hospital malnutrition and cachexia in Slovenia. Eur. J. Clin. Nutr..

[82] Serrano-Urrea R., Garcia-Meseguer M.J. (2013). Malnutrition in an elderly population without cognitive impairment living in nursing homes in Spain: study of prevalence using the Mini Nutritional Assessment test. Gerontology.

[83] Slavikova M. (2018). *Prevalence of malnutrition RISK among institutionalized elderly from north bohemia IS higher than among those in the capital city of PRAGUE, Czech republic.* Central European. J. Publ. Health.

[84] Sahin S. (2016). Prevalence of anemia and malnutrition and their association in elderly nursing home residents. Aging Clin. Exp. Res..

[85] Sharma Y. (2017). Malnutrition in acutely unwell hospitalized elderly - "The skeletons are still rattling in the hospital closet". J. Nutr. Health Aging.

[86] Tsai A.C., Ho C.S., Chang M.C. (2008). Assessing the prevalence of malnutrition with the Mini Nutritional Assessment (MNA) in a nationally representative sample of elderly Taiwanese. J. Nutr. Health Aging.

[87] Tagliaferri S. (2019). The risk of dysphagia is associated with malnutrition and poor functional outcomes in a large population of outpatient older individuals. Clin. Nutr..

[88] Ulger Z. (2013). Malnutrition in Turkish nursing homes: a correlate of short term mortality. J. Nutr. Health Aging.

[89] Vafaei Z. (2013). Malnutrition is associated with depression in rural elderly population. J. Res. Med. Sci..

[90] Vedantam A. (2010). Malnutrition in free-living elderly in rural south India: prevalence and risk factors. Publ. Health Nutr..

[91] van der Sijp M.P.L. (2018). Screening for malnutrition in patients admitted to the hospital with a proximal femoral fracture. Injury-International Journal of the Care of the Injured.

[92] Vanderwee K. (2010). Malnutrition and associated factors in elderly hospital patients: a Belgian cross-sectional, multi-centre study. Clin. Nutr..

[93] Verbrugghe M. (2013). Malnutrition and associated factors in nursing home residents: a cross-sectional, multi-centre study. Clin. Nutr..

[94] Volkert D. (2011). Prevalence of malnutrition in orally and tube-fed elderly nursing home residents in Germany and its relation to health complaints and dietary intake. Gastroenterol Res Pract.

[95] Wong M.M.H. (2019). Malnutrition risks and their associated factors among home-living older Chinese adults in Hong Kong: hidden problems in an affluent Chinese community. BMC Geriatr..

[96] Woo J. (2005). Low staffing level is associated with malnutrition in long-term residential care homes. Eur. J. Clin. Nutr..

[97] Westergren A., Khalaf A., Hagell P. (2015). A Swedish version of the SCREEN II for malnutrition assessment among community-dwelling elderly. Scand. J. Publ. Health.

[98] Win A.Z. (2017). High prevalence of malnutrition among elderly veterans in home based primary care. J. Nutr. Health Aging.

[99] Yoshimura K. (2013). Relationship between depression and risk of malnutrition among community-dwelling young-old and old-old elderly people. Aging Ment. Health.

[100] Zainudin N. (2019). Malnutrition risk and perception on dietary practices among elderly living in agricultural settlements A mixed-methods research. Nutr. Food Sci..

[101] Zenthofer A. (2015). Prosthetic rehabilitation of edentulism prevents malnutrition in nursing home residents. Int. J. Prosthod..

[102] Martin-Sanchez F.J. (2019). Effect of risk of malnutrition on 30-day mortality among older patients with acute heart failure in Emergency Departments. Eur. J. Intern. Med..

[103] Neelemaat F. (2012). Survival of cognitively impaired older hospitalized patients at risk of malnutrition. European Geriatric Medicine.

[104] Nakamura T. (2020). Prognostic impact of malnutrition assessed using geriatric nutritional risk index in patients aged > 80 years with heart failure. Eur. J. Cardiovasc. Nurs..

[105] Tan T. (2016). Identification of comprehensive geriatric assessment based risk factors for malnutrition in elderly asian cancer patients. PLoS One.

[106] Ye X.J. (2018). Comparison of three common nutritional screening tools with the new European Society for Clinical Nutrition and Metabolism (ESPEN) criteria for malnutrition among patients with geriatric gastrointestinal cancer: a prospective study in China. BMJ Open.

[107] Ahmed N. (2018). Impact of malnutrition on survival and healthcare utilization in Medicare beneficiaries with diabetes: a retrospective cohort analysis. Bmj Open Diabetes Research & Care.

[108] de Guzman A.B. (2018). What predicts the malnutrition among a select group of Filipino older persons in institutionalized setting? A partial least square study. Educ. Gerontol..

